# Growth and Chloroplast Replacement of the Benthic Mixotrophic Ciliate *Mesodinium coatsi*

**DOI:** 10.1111/jeu.12709

**Published:** 2019-01-11

**Authors:** Miran Kim, Misun Kang, Myung Gil Park

**Affiliations:** ^1^ Research Institute for Basic Science Chonnam National University Gwangju 61186 Korea; ^2^ LOHABE, Department of Oceanography Chonnam National University Gwangju 61186 Korea

**Keywords:** Acquired phototrophy, *Chroomonas*, cryptophytes, retained chloroplasts, *Rhodomonas*, *Storeatula*

## Abstract

While the ecophysiology of planktonic *Mesodinium rubrum* species complex has been relatively well studied, very little is known about that of benthic *Mesodinium* species. In this study, we examined the growth response of the benthic ciliate *Mesodinium coatsi* to different cryptophyte prey using an established culture of this species. *M. coatsi* was able to ingest all of the offered cryptophyte prey types, but not all cryptophytes supported its positive, sustained growth. While *M. coatsi* achieved sustained growth on all of the phycocyanin‐containing *Chroomonas* spp. it was offered, it showed different growth responses to the phycoerythrin‐containing cryptophytes *Rhodomonas* spp., *Storeatula* sp., and *Teleaulax amphioxeia*. *M. coatsi* was able to easily replace previously ingested prey chloroplasts with newly ingested ones within 4 d, irrespective of prey type, if cryptophyte prey were available. Once retained, the ingested prey chloroplasts seemed to be photosynthetically active. When fed, *M*. *coatsi* was capable of heterotrophic growth in darkness, but its growth was enhanced significantly in the light (14:10 h light:dark cycle), suggesting that photosynthesis by ingested prey chloroplast leads to a significant increase in the growth of *M. coatsi*. Our results expand the knowledge of autecology and ecophysiology of the benthic *M. coatsi*.

THE ciliate *Mesodinium* species inhabit a broad range of aquatic environments, from freshwater to marine ones, and from water column (planktonic) to sandy sediments (benthic), throughout the world. Since the ciliate genus *Mesodinium* was established by Stein in 1863, a total of 10 species have been described to date, including *M. acarus*,* M. fimbriatum*,* M. chamaeleon*,* M. cinctum*,* M. coatsi*,* M. major*,* M. pulex*,* M. pupula*,* M. velox*, and *M. rubrum*; of these, the first two species have been observed in freshwater and the others in marine or estuarine environments (Borror 1972; Dragesco 1963; Foissner et al. 1999; Garcia‐Cuetos et al. 2012; Kahl 1935; Lohmann 1908; Moestrup et al. 2012; Nam et al. 2015; Tamar 1992; Taylor et al. 1971). The taxonomy of *Mesodinium* species has been studied based mainly on morphological characteristics, such as the shape and size of the cell, unique structures of the tentacle, and number of cirri and kinetids (Borror 1972; Dragesco 1963; Kahl 1935; Lohmann 1908; Tamar 1992; Taylor et al. 1971), that have caused the controversy of the genus alternation to *Mesodinium* or *Myrionecta*, as well as ambiguous classification between the species. Phylogenetic studies conducted along with careful ultrastructural observations, however, have clarified the phylogenetic positions of at least six *Mesodinium* species, including *M. pulex*,* M. pupula*,* M. rubrum*,* M. major*,* M. chamaeleon*, and *M. coatsi*, the latter three of which have only recently been added to the genus (Garcia‐Cuetos et al. 2012; Moestrup et al. 2012; Nam et al. 2015).

Among *Mesodinium* species, the ecophysiology of the planktonic species *M. rubrum* has been the best studied for a long time. It is well known as a species that causes massive, nontoxic blooms in coastal and estuarine areas worldwide (Crawford 1989; Lindholm 1985; Taylor et al. 1971). Furthermore, early ultrastructural studies of *M. rubrum* isolated from field samples reported that the cells of this ciliate contained chloroplasts, mitochondria, and nucleomorphs originating from its cryptophyte prey (Gustafson et al. 2000), which were previously thought to represent incomplete endosymbionts (Hibberd 1977; Oakley and Taylor 1978; Taylor et al. 1969, 1971). Since cultures of *M. rubrum* have been established (Gustafson et al. 2000; Yih et al. 2004), however, it has been revealed that *M. rubrum* acquires functional chloroplasts, nuclei, and other cellular organelles from its cryptophyte prey, which mainly belong to the genera *Geminigera* and *Teleaulax*, in order to maintain stable photosynthetic performance and growth (Hansen et al. 2012; Johnson and Stoecker 2005; Johnson et al. 2006, 2007; Kim et al. 2016, 2017). For this reason, *Mesodinium* species has come into the spotlight as one of the model organisms from which clues to the evolutionary history of plastid acquisition may be drawn. However, very little is known about such ecophysiological features as trophic level, feeding behavior and process, and chloroplast retention and function of *Mesodinium* species other than *M. rubrum*. Only *M. pulex* and *M. chamaeleon*, which are both benthic species, have been studied from the establishment of culture, and growth and grazing responses, and chloroplast dynamics of the latter species has recently been reported (Jakobsen et al. 2006; Johnson et al. 2004; Moeller and Johnson 2017; Moestrup et al. 2012; Tarangkoon and Hansen 2011). However, the amount of ecophysiological information available for other benthic *Mesodinium* species still remains insufficient.

We have been maintaining a culture of the benthic species *Mesodinium coatsi* by providing it with benthic cryptophyte *Chroomonas* species as prey. Using these cultures of *M. coatsi* and its cryptophyte prey, in this study we investigated: (1) the effects of different cryptophyte prey on the growth of *M. coatsi*; (2) the replacement of ingested prey chloroplasts within *M. coatsi*; and (3) the growth responses of fed *M. coatsi* in both light/dark conditions and total darkness. Through this work, we provide new insights into the basic autecology and ecophysiology of the benthic species *M. coatsi*.

## Materials and Methods

### Culture of the benthic ciliate *Mesodinium coatsi*

A culture of *M. coatsi* (MC01‐LOHABE) was established by isolating single cells from samples collected from the sandy sediments of Mohang beach (35°34′N, 126°30′E), Korea, on October 17, 2011 (Nam et al. 2015). Briefly, surface sand samples were collected with a spoon during low tide, and then were transported directly to the laboratory. Individual ciliate cells were isolated using a drawn glass pipette, washed eight times in sterile seawater, and transferred to a 24‐well plate (SPL Lifesciences, Gyeonggido, Korea) containing 1 ml of 30 psu f/2‐Si medium (+ 5% v/v soil extract) at 20 °C under a 14:10 h light:dark cycle with cool‐white fluorescent light at 80 μmol photons/m^2^/s. Light intensity was measured with a photometer (Biospherical Instruments, Model QSL‐2101, San Diego, CA). *M. coatsi* culture has since been maintained in three stock cultures by separately adding the marine benthic cryptophytes *Chroomonas* sp. 07 (gCR07‐LOHABE), *Chroomonas* sp. 09 (gCR09‐LOHABE), and *Chroomonas* sp. 12 (gCR12‐LOHABE) as prey over the past 7 years. Prey were supplied and replenished whenever they were depleted in the cultures. Once *M. coatsi* cell density increased, the cells were transferred to a 125 ml KIMAX^®^ glass culture flask (KIMBLE, Vineland, NJ) and were kept well‐fed by adding sufficient amounts of prey. All of the cultures used in this study were non‐axenic.

### Cultures of cryptophytes

**Figure 1:**
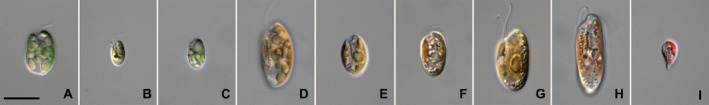
Light micrographs of cryptophyte species used in this study. (**A**) *Chroomonas* sp. 07 (gCR07‐LOHABE), (**B**) *Chroomonas* sp. 09 (gCR09‐LOHABE), (**C**) *Chroomonas* sp. 12 (gCR12‐LOHABE), (**D**) *Rhodomonas* sp. 01 (rCR01‐LOHABE), (**E**) *Rhodomonas* sp. 02 (rCR02‐LOHABE), (**F**) *Rhodomonas* sp. 03 (rCR03‐LOHABE), (**G**) *Rhodomonas* sp. 04 (rCR04‐LOHABE), (**H**) *Storeatula* sp. (CCMP1868), (**I**) *Teleaulax amphioxeia* (CR01‐LOHABE). Chloroplasts in the former three species have phycocyanin pigments, while those in the latter six species have phycoerythrin pigments. The scale bar in A is 10 μm and applies to all panels.

A total of nine cryptophyte strains were used as prey for the experiment in this study (Table 1; Fig. 1). The benthic cryptophytes *Chroomonas* sp. 07 (gCR07‐LOHABE), *Chroomonas* sp. 09 (gCR09‐LOHABE), *Chroomonas* sp. 12 (gCR12‐LOHABE), and *Rhodomonas* sp. 04 (rCR04‐LOHABE) were isolated in September 2011 from the same site where *M. coatsi* was isolated. The benthic cryptophytes *Rhodomonas* sp. 01 (rCR01‐LOHABE), *Rhodomonas* sp. 02 (rCR02‐LOHABE), and *Rhodomonas* sp. 03 (rCR03‐LOHABE) were isolated from sand samples taken from Jinhae and Dongho, Korea, in August and September 2011, respectively. For the isolation of benthic cryptophytes, surface sand samples were collected with a spoon during low tide and transported directly to the laboratory. The samples were observed under an inverted IX51 microscope (Olympus IX51, Tokyo, Japan). Individual cells were isolated using a drawn glass pipette, washed five times in sterile seawater, and then transferred to a 24‐well plate (SPL Lifesciences, Gyeonggido, Korea) containing 1 ml of 30 psu f/2‐Si medium (+ 5% v/v soil extract). The planktonic cryptophyte *T. amphioxeia* (CR01‐LOHABE) was isolated from surface water collected in Masan Bay, Korea, in November 2007. All cryptophyte cultures were grown under the same conditions described above. Once cell density in cultures increased, the cells were transferred to 125 ml KIMAX^®^ glass culture flasks and were maintained as stock cultures.

**Table 1:** Summary of nine cryptophytes offered as potential prey for *Mesodinium coatsi* in growth response experiment

Species	Strain name	Location	Isolate Date	Life style	Chloroplast color	Cell size ±SE (μm)		Ingestion (Y/N)
						Length	Width	
*Chroomonas* sp. 07	gCR07‐LOHABE	Buan, Korea	Sep. 08. 2011	Benthic	Green	12.7 ± 0.3	7.5 ± 0.3	Y
*Chroomonas* sp. 09	gCR09‐LOHABE	Buan, Korea	Sep. 02. 2011	Benthic	Green	7.4 ± 0.2	5.2 ± 0.2	Y
*Chroomonas* sp. 12	gCR12‐LOHABE	Buan, Korea	Sep. 02. 2011	Benthic	Green	7.9 ± 0.1	4.7 ± 0.1	Y
*Rhodomonas* sp. 01	rCR01‐LOHABE	Jinhae, Korea	Aug. 06. 2011	Benthic	Reddish‐brown	18.9 ± 0.7	9.2 ± 0.4	Y
*Rhodomonas* sp. 02	rCR02‐LOHABE	Gochang, Korea	Sep. 22. 2011	Benthic	Reddish‐brown	10.4 ± 0.2	6.0 ± 0.1	Y
*Rhodomonas* sp. 03	rCR03‐LOHABE	Gochang, Korea	Sep. 22. 2011	Benthic	Reddish‐brown	12.6 ± 0.2	8.2 ± 0.3	Y
*Rhodomonas* sp. 04	rCR04‐LOHABE	Buan, Korea	Sep. 16. 2011	Benthic	Reddish‐brown	14.8 ± 0.4	7.8 ± 0.1	Y
*Storeatula* sp.	CCMP1868	Prakeet Bay, Australia	–	Benthic	Reddish‐brown	19.1 ± 0.5	9.4 ± 0.1	Y
*Teleaulax amphioxeia*	CR01‐LOHABE	Masan, Korea	Nov. 03. 2007	Planktonic	Light‐red	7.6 ± 0.3	4.7 ± 0.1	Y

N = no; Y = yes

### Growth responses to different cryptophytes

These experiments were performed to investigate the growth responses of *M*. *coatsi* to provided different cryptophyte prey. *M.** **coatsi* cells originally grown on *Chroomonas* sp. 09 and then starved for 2 d were used for the following two experiments (1 and 2). Prior to the experiment, the absence of prey in the culture was confirmed under an inverted microscope at 100X magnification (Olympus IX51).

#### Experiment 1: *Chroomonas* spp. and *Teleaulax amphioxeia* as prey

Three benthic cryptophytes, *Chroomonas* spp. 07, 09, and 12, as well as the planktonic cryptophyte *T. amphioxeia* were used as prey for *M.** **coatsi*. Experiment 1 was performed on well plates to allow for the precise enumeration of *Chroomonas* spp. cells due to the strong adhesion of these prey types to the substrate. The initial predator:prey ratio was adjusted to achieve a ratio of 1:10, with concentrations of *M.** **coatsi* and each prey cell being about 20 and 200 cells/ml, respectively. Mixtures of prey and predator (1 ml) were distributed among three wells, and either prey‐only or predator‐only controls were also respectively established in three additional wells (in triplicate) of a 48‐well plate. Each plate in all treatments and controls was fixed by adding 20 μL of Lugol's solution (final concentration 2%) into each well at 2‐ to 4‐d intervals for 14 d. Whole *M.** **coatsi* cells in the well were counted directly under an inverted microscope (Olympus IX51), whereas abundances of different cryptophyte prey were enumerated using a Palmer–Maloney chamber. Unfortunately, unlike other cryptophyte prey, the enumeration of *Chroomonas* sp. 12 cells could not be done at each sampling time because of their aggregative characteristics.

#### Experiment 2: *Rhodomonas* spp. and *Storeatula* sp. as prey

Five benthic cryptophytes, *Rhodomonas* spp. 01, 02, 03, and 04, and *Storeatula* sp. (CCMP1868), were used as prey for *M.** **coatsi*. The initial predator:prey ratio was adjusted to achieve ratios from 1:2 to 1:5, depending on the type of cryptophyte prey used. Mixtures of prey and predator were distributed among three culture flasks, and either prey‐only or predator‐only controls were also respectively established in three additional culture flasks. Aliquots (2 ml) withdrawn from each flask at 1‐ to 2‐d intervals over 15 d were fixed with acid Lugol's solution (final concentration 2%). All experimental cultures (from experiments 1 and 2) were placed on a shelf under the same culture condition described above. The abundances of *M.** **coatsi* and different cryptophytes were enumerated using a Sedgewick‐Rafter chamber under the inverted microscope (Olympus IX51) at 100X and 200X. Growth rates of *M.** **coatsi* (μ) provided with different cryptophyte prey were calculated using the exponential growth equation, μ = ln(*N*_2_/*N*_1_)/(*t*_2_ ****− *t*_1_), where *N*_2_ and *N*_1_ are cell concentrations at time *t*_2_ and time *t*_1_, respectively, and *t*_2_ ****− *t*_1_ is the time interval between samplings. The time interval (*t*_2_ ****− *t*_1_) was selected only for the exponential phase, mostly 2 d after the start of experiment 1 and the day after abundance of non‐fed *M.** **coatsi* (control) began to decrease in experiment 2. The ingestion rates were calculated according to the method of Jeong and Latz (1994).

### Cross‐feeding experiment: chloroplasts turnover

The aim of this experiment was to examine how fast the retained prey chloroplasts are replaced with new ones from recently ingested prey. To track chloroplast replacement easily and clearly, two cryptophytes with differently colored chloroplasts (the green chloroplasts of *Chroomonas* sp. 09 and reddish‐brown chloroplasts of *Rhodomonas* sp. 03 on which *M.** **coatsi* grew best in the prior experiments 1 and 2) were used as chloroplast donors. *M.** **coatsi* cells initially grown on *Chroomonas* sp. 09 and then starved for 2 d were allowed to feed on *Rhodomonas* sp. 03 for 9 d in a culture flask (Corning Inc., Corning, NY, USA) at a predator:prey ratio of approximately 1:3. Two days after *Rhodomonas* sp. 03 was depleted in the culture, *M.** **coatsi* cells were offered back *Chroomonas* sp. 09 as prey for 8 d. When the prey (*Rhodomonas* sp. 03 and *Chroomonas* sp. 09) concentration was equal to or less than that of the predator, additional prey were provided to maintain sufficient prey concentrations during the experiment. Two aliquots (1 ml and 2 ml) withdrawn from the flask were fixed with 25% glutaraldehyde (final concentration 1%) and acid Lugol's solution, respectively. *M.** **coatsi* cells fixed with glutaraldehyde were observed under the green‐light excitation setting of an inverted microscope at 200X magnification (Carl Zeiss Axio Vert. A1, Hallbergmoos, Germany) to allow for the clear distinction of the two different chloroplast types within the cells. The first 100 *M.** **coatsi* cells encountered were examined in each sample. The orange‐fluorescing chloroplasts were regarded as the chloroplasts of *Rhodomonas* sp. 03, while the red‐fluorescing chloroplasts were regarded as the chloroplasts of *Chroomonas* sp. 09. Abundances of *M.** **coatsi* and their prey were enumerated using a Sedgewick‐Rafter chamber under an inverted microscope (Olympus IX51) at 100X and 200X from Lugol's solution‐fixed samples.

### Growth responses of *Mesodinium coatsi* in both light/dark and dark conditions

Growth responses of *Mesodinium coatsi* growing in mixotrophic cultures (i.e. *M.** **coatsi* offered either *Chroomonas* sp. 09 or *Rhodomonas* sp. 03) were conducted in both light/dark and dark conditions to investigate whether the mixotrophic growth of *M.** **coatsi* was dependent on light or purely ingestion of prey. Prior to the experiment, the absence of prey in the *M.** **coatsi* culture was confirmed under an inverted microscope at 100X magnification (Olympus IX51). *M.** **coatsi* cells that had been starved for 3 d were supplied with either *Chroomonas* sp. 09 or *Rhodomonas* sp. 03 as prey at a predator:prey ratio of approximately 1:25 or 1:10, respectively, in two sets of triplicate culture flasks (Corning Inc.) containing f/2‐Si medium for both light/dark and complete darkness treatments. Parallel predator‐ and prey‐only controls were also established, consisting of only *M. coatsi* cells and only *Chroomonas* sp. 09 or *Rhodomonas* sp. 03, respectively. All experimental cultures in the light/dark condition were kept at the same conditions described above, but for the dark treatment experimental culture flasks were kept in complete darkness by wrapping them in aluminum foil. Subsamples (2 ml) taken from each flask every day over 6 d were fixed with acid Lugol's solution (final concentration 2%) for cell enumeration in a Sedgewick‐Rafter chamber under an inverted microscope (Olympus IX51) at 100X and 200X.

### Light microscopy

Light micrographs of live *M. coatsi* and cryptophyte prey were taken at 1,000X magnification using a photomicrographic system (AxioCam HRC, Carl Zeiss Inc.) coupled to a bright‐field microscope equipped with differential interference contrast optics (Axio imager A2; Carl Zeiss Inc.).

### DNA extraction, PCR amplification, and sequencing

Aliquots (3 ml) withdrawn from each cryptophyte culture were subjected to centrifugation for 5 min at 7168 *g*. The DNA was then extracted and purified using the Genomic DNA Extraction Kit (Bioneer, Daejeon, Korea). Gene fragments of cryptophyte nuclear SSU rRNA were amplified by polymerase chain reaction (PCR) using the common primer set (EukA/EukB) or a newly designed primer set (GCRSSU‐F/GCRSSU‐R) (Table 2). PCR conditions were as follows: 94 °C for 5 min followed by 40 cycles of 94 °C for 45 s, 55 °C for 30 s, and 72 °C for 80 s followed by 72 °C for 10 min. A semi‐nested PCR was also conducted for the detection of gene fragments of *Rhodomonas* and *Storeatula* species using a second pair of primers, 18SNF2 and EukB. For this semi‐nested PCR, the first PCR product was initially amplified using the universal primer set for 20 cycles. Subsequently, 3 μl of the first PCR product was then used as a template and run through the amplification again, as described above. The PCR products were visualized through EcoDye™ (SolGent, Daejeon, Korea) stained 1% agarose gel electrophoresis and purified using a PCR purification kit (Bioneer). The amplified DNA was sequenced using an ABI3730xl DNA Analyzer at the Macrogen sequencing service (Macrogen Inc., Daejeon, Korea). Sequences were edited and assembled into contigs using ContigExpress (Vector NTI version10.1; Invitrogen, Carlsbad, CA, USA). The nuclear SSU rRNA gene sequences of the nine cryptophytes used in this study have been deposited in Genbank under the following accession numbers: *Chroomonas* sp. 07 (gCR07‐LOHABE) (MG196041), *Chroomonas* sp. 09 (gCR09‐LOHABE) (MG196042), *Chroomonas* sp. 12 (gCR12‐LOHABE) (MG196043), *Rhodomonas* sp. 01 (rCR01‐LOHABE) (MH107134), *Rhodomonas* sp. 02 (rCR02‐LOHABE) (MG196040), *Rhodomonas* sp. 03 (rCR03‐LOHABE) (MH107145), *Rhodomonas* sp. 04 (rCR04‐LOHABE) (MH107133), *Storeatula* sp. CCMP1868 (MH108117), and *T.** **amphioxeia* (CR01‐LOHABE) (MH107135).

**Table 2:** Primers used and newly designed in this study for amplifying the nuclear SSU rRNA gene of cryptophytes

Primer	Sequence (5′–3′)	Target species	Source
1st			
GCRSSU‐F GCRSSU‐R	TGC CAG TAG TCA TAY GCT TGT CTY TGC AGG TTC ACY TAC GGA AA	*Chroomonas* sp. 07 (gCR07‐LOHABE) *Chroomonas* sp. 12 (gCR12‐LOHABE)	This study
EukA EukB	AAC CTG GTT GAT CCT GCC AGT TGA TCC TTC TGC AGG TTC ACC TAC	*Chroomonas* sp. 09 (gCR09‐LOHABE) *Teleaulax amphioxeia* (CR01‐LOHABE)	Medlin et al. (1988)
2nd			
18SNF2	TGA TGG TCC CTT ACT ACA	*Rhodomonas* sp. 01 (rCR01‐LOHABE) *Rhodomonas* sp. 02 (rCR02‐LOHABE) *Rhodomonas* sp. 03 (rCR03‐LOHABE) *Rhodomonas* sp. 04 (rCR04‐LOHABE) *Storeatula* sp. (CCMP1868)	Majaneva et al. (2014)

### Phylogenetic analyses

The alignment of each cryptophyte species’ nuclear gene sequences was constructed using the Clustal X algorithm and refined by eye using the Genetic Data Environment (GDE 2.4) program (Smith et al. 1994). A maximum likelihood (ML) tree with 2,000 bootstrap replicates was inferred for each alignment using RAxML version 8 (Stamatakis 2014). Prior to Bayesian analysis, we performed a likelihood ratio test using Modeltest, version 3.7 (Posada and Crandall, 1998) to determine the best model for the combined dataset. Bayesian analysis was run using MrBayes 3.2.5 (Ronquist et al. 2012), with four Metropolis‐coupled Markov chain Monte Carlo (MCMC) processes run for 20,000,000 generations, keeping one tree every 1,000 generations. The data were analyzed with a single GTR + I + G model and the following parameters were specified: Prset revmatpr = dirichlet (1.3820, 3.4431, 1.1997, 1.9270, 6.5590, 1.0); statefreqpr = dirichlet (0.2779, 0.1934, 0.2571, 0.2717); shapepr = exponential (0.6718); and pinvarpr = fixed (0.5991) for the nuclear SSU rRNA genes. The first 8000 trees were discarded as burn‐in. Trees were visualized using the Figtree v.1.4.2 program.

## Results

### Phylogenetic positions of the cryptophytes used in this study

**Figure 2:**
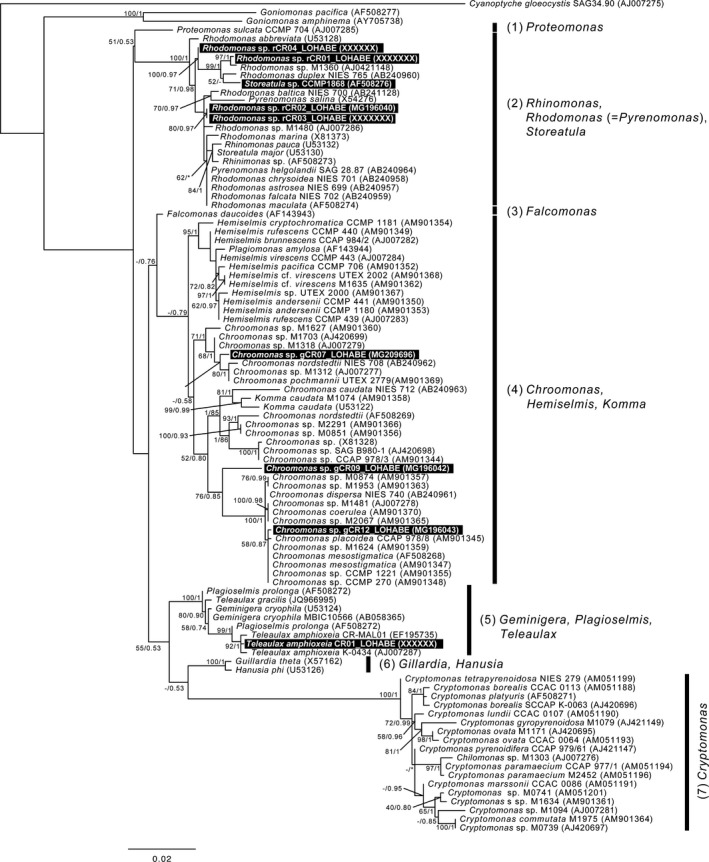
The phylogenetic tree of cryptophytes inferred from Bayesian analysis and RAxML based on nuclear SSU rDNA sequences in this study. The sequences of the nine cryptophytes determined in this study are indicated by black boxes. At internodes, tree support values are represented in terms of both posterior probabilities and bootstrap values, respectively. Values of <0.5 or 50% are shown as “−”. Mismatches of the branch between the Bayesian and RAxML tree are indicated by a “*”.

Cryptophytes, including the nine experimental strains used in this study, were divided into seven distinct clades in the phylogenetic tree derived from the analysis of their nuclear SSU rRNA genes (Fig. 2), including two clades comprising the monospecific genera *Proteomonas* (clade 1) and *Falcomonas* (clade 3). *Rhodomonas* spp. 01, 02, 03, and 04, and *Storeatula* sp., were included in clade 2, and *Chroomonas* spp. 07, 09, and 12 were placed in clade 4. The planktonic *T. amphioxeia* was included in clade 5. In the nuclear SSU rRNA‐based phylogeny, all cryptophyte prey used in this study were placed in different phylogenetic positions, except for *Rhodomonas* spp. 02 and 03, which were in the same position.

### Effects of different cryptophyte prey on the growth of *Mesodinium coatsi*

**Figure 3:**
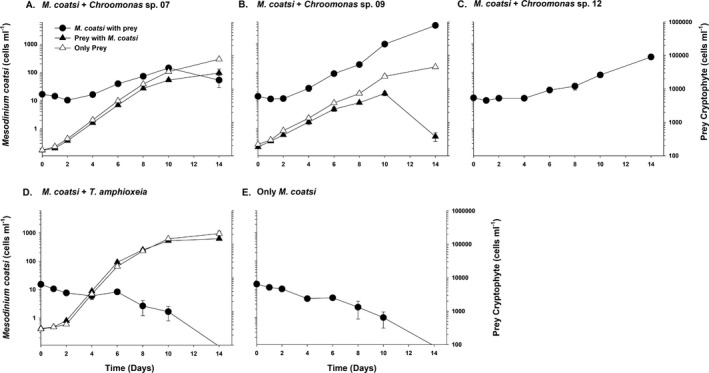
Growth responses of the benthic ciliate *Mesodinium coatsi* when offered four different cryptophytes (three phycocyanin‐containing *Chroomonas* spp. and one phycoerythrin‐containing *Teleaulax amphioxeia*) as prey: (**A**) *Chroomonas* sp. 07 (gCR07‐LOHABE), (**B**) *Chroomonas* sp. 09 (gCR09‐LOHABE), (**C**) *Chroomonas* sp. 12 (gCR12‐LOHABE), and (**D**) *Teleaulax amphioxeia* (CR01‐LOHABE). (**E**) Growth response of *M. coatsi* without prey. Symbols and error bars indicate mean values and standard errors of triplicate cultures, respectively.

**Figure 4:**
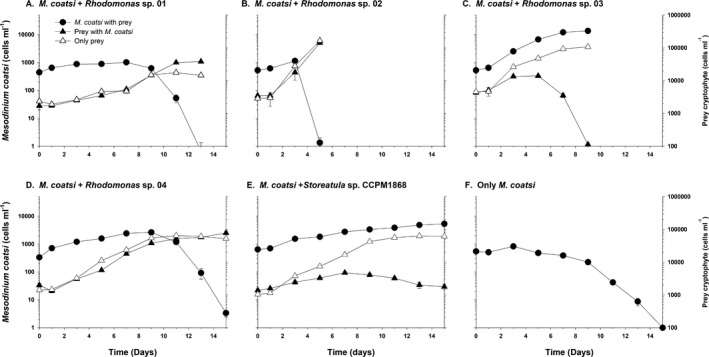
Growth responses of the benthic ciliate *Mesodinium coatsi* when offered five different, phycoerythrin‐containing cryptophytes as prey: (**A**) *Rhodomonas* sp. 01 (rCR01‐LOHABE), (**B**) *Rhodomonas* sp. 02 (rCR02‐LOHABE), (**C**) *Rhodomonas* sp. 03 (rCR03‐LOHABE), (**D**) *Rhodomonas* sp. 04 (rCR04‐LOHABE), and (**E**) *Storeatula* sp. (CCMP1868). (**F**) Growth response of *M. coatsi* without prey. Symbols and error bars indicate mean values and standard errors of triplicate cultures, respectively.

**Figure 5:**
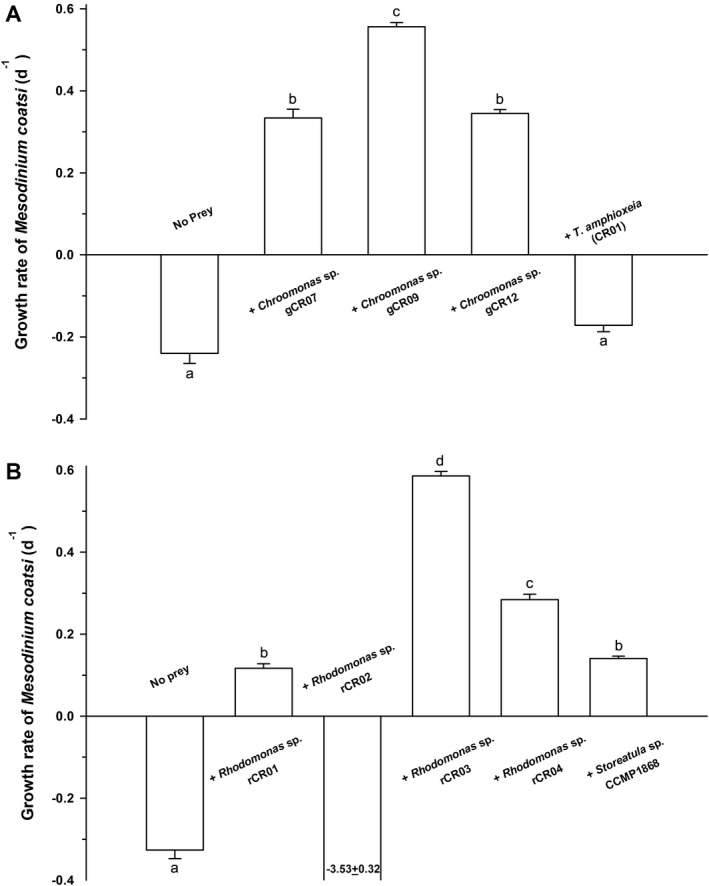
Growth rates of the benthic ciliate *Mesodinium coatsi* when fed different cryptophytes (**A**) *Chroomonas* spp. and *Teleaulax amphioxeia*, (**B**) *Rhodomonas* spp. and *Storeatula* sp. Growth rates were calculated from the experiments in Fig. 3 and 4. Bars are means ± SE for triplicate cultures. Note that while all offered *Chroomonas* spp. supported considerable growth of *M. coatsi*, only *Rhodomonas* sp. 03 supported the significant positive growth of *M. coatsi*. The other *Rhodomonas* spp. and *Storeatula* sp. allowed for relatively lower growth than the four‐species mentioned above. Negative growth was found when the ciliate was exposed to *T. amphioxeia* and *Rhodomonas* sp. 02. Lower case letters on A and B indicate significant differences among treatments at the *P *<* *0.05 level (one‐way ANOVA, Tukey's HSD test). An erroneous negative growth rate, which was obtained when the ciliate was fed *Rhodomonas* sp. 02, was excluded in the statistical test shown in B.

*Mesodinium coatsi* was able to ingest all offered cryptophyte prey (Movies [Link], [Link], [Link], [Link], [Link], [Link], [Link], [Link], [Link]), but its growth responses to different cryptophytes were different (Fig. 3, 4, 5). *M. coatsi* exhibited exponential growth when offered all three *Chroomonas* spp. (07, 09, and 12), and *Rhodomonas* sp. 03 (Fig. 3A–C, 4C). In the first experiment (i.e. *M. coatsi* fed *Chroomonas* spp. and *T. amphioxeia* as prey), the highest growth rate (0.56 ± 0.01 d^−1^) (mean ± SE) was obtained when *M. coatsi* was fed *Chroomonas* sp. 09, and this was significantly higher than those of the ciliate when it was fed *Chroomonas* sp. 07 (0.33 ± 0.02 d^−1^) or *Chroomonas* sp. 12 (0.34 ± 0.01 d^−1^) (one‐way ANOVA, Tukey's HSD test, *P *<* *0.001). *M. coatsi* exposed to *T*. *amphioxeia* failed to show a sustainable growth (Fig. 3D). The growth rate of *M. coatsi* fed on *T. amphioxeia* was −0.17 (±0.02) d^−1^, which was similar to that (−0.24 ± 0.02 d^−1^) of *M. coatsi* without prey (*P *>* *0.05). In the second experiment (i.e. *M. coatsi* fed *Rhodomonas* spp. and *Storeatula* sp. as prey), a sustained high growth rate (0.59 ± 0.01 d^−1^) was only achieved when the ciliate was fed *Rhodomonas* sp. 03 (Fig. 4C, 5B). *M. coatsi* numbers increased temporarily after feeding on *Rhodomonas* spp. 01 (0.12 ± 0.01 d^−1^) and 04 (0.28 ± 0.01 d^−1^), and *Storeatula* sp. (0.14 ± 0.01 d^−1^), but these ingestions were not linked to sustained, longer term growth (Fig. 4A, D, E). The abundance of *M. coatsi* offered *Rhodomonas* sp. 02 increased temporarily during the first 3 d and then sharply dropped to zero (Fig. 4B). The ingestion rates of *M. coatsi* on *T. amphioxeia* and *Rhodomonas* sp. 02 were 0.03 ± 0.01 ng C/predator/d, 0.02 ± 0.01 ng C/predator/d (mean ± SE), respectively, and were not significantly different from zero (*t*‐test, *P *>* *0.5).

### Chloroplast replacement

**Figure 6:**
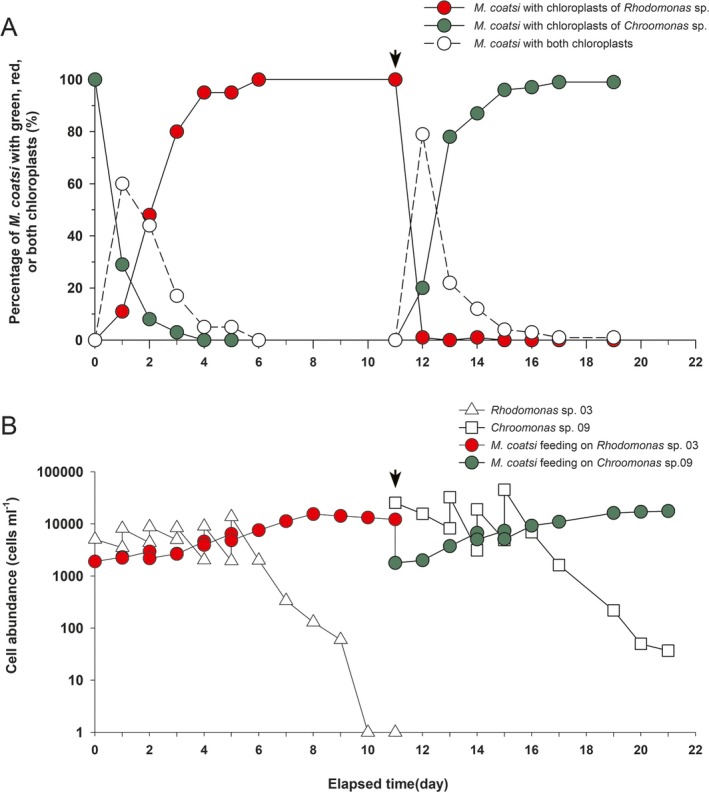
Cross‐feeding experiment examining chloroplast replacement in the benthic ciliate *Mesodinium coatsi*. *M*.* coatsi* grown on *Chroomonas* sp. 09 were offered *Rhodomonas* sp. 03 at the start of the experiment, and later switched again to being offered *Chroomonas* sp. 09 on Day 11. The chloroplasts of *Rhodomonas* sp. 03 and *Chroomonas* sp. 09 within *M. coatsi* are represented as red and blue, respectively, in (**A**). A. Percentage of *M. coatsi* with green, red, or both chloroplasts as a function of elapsed time. (**B**) Abundances of *M. coatsi* and its cryptophyte prey, *Rhodomonas* sp. 03 and *Chroomonas* sp. 09. Arrows indicate the point at which the prey type supplied was switched.

When sufficient numbers of *Rhodomonas* sp. 03 were supplied as prey (Fig. 6B), the chloroplasts of most *M. coatsi* cells (98%) completely switched from the old, green chloroplasts of *Chroomonas* sp. 09 to the new, reddish‐brown chloroplasts of *Rhodomonas* sp. 03 within 4 d (Fig. 6A). Some *M. coatsi* cells (11%) were even able to entirely exchange all of their old chloroplasts with new ones from recently ingested prey within a single day. After 6 d, the chloroplasts from the previous prey (*Chroomonas* sp. 09) were completely replaced with those of new prey (*Rhodomonas* sp. 03). The chloroplast turnover time was similar in the reverse situation. When *M. coatsi* cells starved for 2 d while retaining chloroplasts of *Rhodomonas* sp. 03 were exposed to *Chroomonas* sp. 09 again, the chloroplasts of most *M. coatsi* cells (96%) were again replaced with those of *Chroomonas* sp. 09 within 4 d (Fig. 6A).

### Mixotrophic growth of *Mesodinium coatsi* in both light/dark and dark conditions

**Figure 7:**
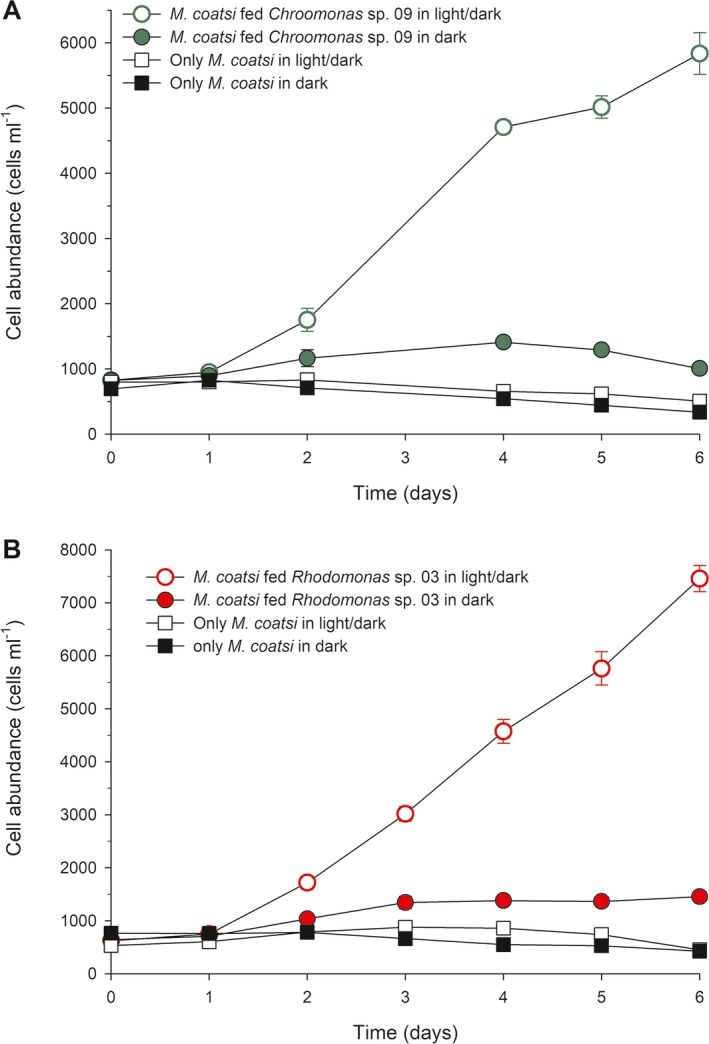
Growth responses of the *Mesodinium coatsi* to the presence of cryptophyte prey in both light/dark (14:10 h) and dark conditions. (**A**) Abundances of *M. coatsi* fed *Chroomonas* sp. 09 and non‐fed *M. coatsi* as function of light. (**B**) Abundances of *M. coatsi* fed *Rhodomonas* sp. 03 and non‐fed *M. coatsi* as function of light. Note that growth of *M. coatsi* was significantly enhanced in light compared to that in total darkness, irrespective of prey type. Data points are shown as mean ± SE for triplicate cultures.

*Mesodinium coatsi* abundance greatly increased after feeding on prey (*Chroomonas* sp. 09 or *Rhodomonas* sp. 03), but its growth responses differed markedly as a function of light (Fig. 7). In the light/dark condition, the growth of *M. coatsi* in the presence of either *Chroomonas* sp. 09 or *Rhodomonas* sp. 03 was considerably enhanced relative to that in total darkness (one‐way ANOVA, Tukey's HSD test, *P *<* *0.005), with growth rates in light/dark conditions being 0.54 (±0.03) d^−1^ and 0.60 (±0.02) d^−1^ on each respective prey type, compared to 0.15 (±0.02) d^−1^ and 0.22 (±0.01) d^−1^ in equivalent conditions under darkness. In darkness, *M. coatsi* in the presence of prey (whether *Chroomonas* sp.09 or *Rhodomonas* sp. 03) showed positive growth, whereas in the absence of prey it showed no or negative growth.

## Discussion

### Prey specificity of *Mesodinium coatsi*

The ciliate *Mesodinium* species are phagotrophs that rely on prey ingestion for growth (Gustafson et al. 2000; Tarangkoon and Hansen 2011; Yih et al. 2004). The prey specificity of some *Mesodinium* species has been explored in previous studies. The mixotrophic species *M. rubrum* is known to feed on cryptophytes belonging to the *Teleaulax*/*Plagioselmis*/*Geminigera* (TPG) clade (Gustafson et al. 2000; Hansen and Fenchel 2006; Johnson and Stoecker 2005; Yih et al. 2004), but it prefers to feed on a certain cryptophyte species so that it can undergo sustained growth. The temperate *M. rubrum* showed an extraordinarily high growth rate when fed *T. amphioxeia*–like species compared to when it was fed other TPG cryptophytes (Hansen et al. 2012; Johnson et al. 2016; Myung et al. 2011; Park et al. 2007; Raho et al. 2014), which is consistent with field observations of this species. Most natural *M. rubrum* cells have been reported to predominately retain *T. amphioxeia*–like plastids (Herfort et al. 2011; Johnson et al. 2016; Nishitani et al. 2010). While *M. rubrum* has a relatively narrow prey range, the benthic species *M. pulex* feeds on a wide range of prey organisms, including the cryptophytes *Guilardia theta*,* Rhodomonas* sp., *Teleaulax* sp., and *T. amphioxeia*, as well as the dinoflagellate *Heterocapsa rotundata*, but its growth differs depending on what prey organisms it ingests (Johnson et al. 2004; Tarangkoon and Hansen 2011). The mixotroph *M. chamaeleon* has also been reported to ingest prey belonging to at least five different genera of cryptophytes (i.e. *Chroomonas mesostigmatica*,* Guillardia theta*,* Hemiselmis cryptochromatica*,* Storeatula major*, and *Teleaulax amphioxeia*), but it showed a distinct prey preference for *Storeatula major* (Moeller and Johnson 2017; Moestrup et al. 2012), which is smaller than the *Storeatula* sp. CCMP1868 strain used in our study.

Our study showed that *M. coatsi* can, although temporarily, feed on a relatively broad array of cryptophyte prey, similar to *M. chamaeleon*. *M. coatsi* was also able to ingest phylogenetically distinct prey belonging to four different cryptophyte genera (*Chroomonas*,* Rhodomonas*,* Storeatula,* and *Teleaulax*), but not all of these ingestions have supported its sustained growth and thus its growth responses varied as a function of the prey species it consumed. These results may be due to several factors. It seems unlikely that prey size would affect the observed differences in growth responses because *M. coatsi* is capable of ingesting prey of various cell sizes, ranging from 7.4 (*Chroomonas* sp. 09) to 19.1 μm (*Storeatula* sp. CCMP1868), using its retractable‐extensible tentacles. Rather, it is likely that the differential growth responses were related to differences in habitat between the predator and prey organisms. For example, *M. coatsi* did not grow when offered *T. amphioxeia,* and eventually died out. *T. amphioxeia* is planktonic and prevalent throughout the water column (Herfort et al. 2011; Johnson et al. 2016; Peterson et al. 2013), whereas *M. coatsi* mainly inhabits benthic sedimentary environments. Such a spatial separation may have caused some of the difficulty for *M. coatsi* in exploiting the planktonic *T. amphioxeia* due to low natural encounter rates between them, which may in turn, if any, have resulted in the ingestion of *T. amphioxeia* by *M. coati* not at rates that have any effect on the population dynamics of planktonic cryptophyte prey, as indicated from the near‐zero level of the ingestion rate. Third, chloroplast type of its cryptophyte prey may also have a significant effect on the growth of *M. coatsi*. It is known that cryptophytes with green or blue‐green chloroplasts (e.g. *Chroomonas, Hemiselmis,* and *Komma*) possess phycocyanin pigments, while those with red or reddish‐brown chloroplasts (e.g. *Geminigera, Guillardia, Hanusia, Plagioselmis, Rhinomonas, Rhodomonas, Storeatula,* and *Teleaulax*) have phycoerythrin pigments (Gantt 1979; Gantt et al. 1971; Hoef‐Emden 2008). In our experiment, *M. coatsi* achieved sustained growth on all phycocyanin‐containing *Chroomonas* spp., whereas the growth responses of *M. coatsi* fed *Rhodomonas* spp. and *Storeatula* sp., which have chloroplasts with phycoerythrin pigments, were distinct and variable. While *Rhodomonas* sp. 03 supported a growth rate of *M. coatsi* as high as that with *Chroomonas* spp., *Rhodomonas* spp. 01 and 04, and *Storeatula* sp. (CCMP1868), did not support sustained growth in the long run, although they did temporarily support the growth of *M. coatsi*. Given that *Rhodomonas* spp. 02 and 03 occupy the same phylogenetic position and have similar cell sizes, it is noteworthy that *M. coatsi* showed opposite growth responses to these two species, although the reason for this currently remains unknown. Garcia‐Cuetos et al. (2012) and Moeller and Johnson (2017) observed that most benthic *Mesodinium* species frequently retain green chloroplasts in nature, indicating that such species, including *M. chamaeleon* and *M. coatsi,* may prefer to feed on phycocyanin‐containing cryptophytes over phycoerythrin‐containing cryptophytes in their benthic environments. Nonetheless, we have occasionally observed *M. chamaeleon* or *M. coatsi*‐like species containing both green and reddish‐brown chloroplasts in field samples (M. Kim, pers. observ.). Along with our field observations, the remarkable growth of *M. coatsi* on *Rhodomonas* sp. 03 in our laboratory experiment suggests that *M. coatsi* can also exploit some phycoerythrin‐containing members of the *Rhinomonas*/*Rhodomonas*/*Storeatula* clade, depending on the composition of the benthic cryptophyte prey community.

### Chloroplast replacement

We confirmed that *Mesodinium coatsi* can replace the chloroplasts of its previous prey with those from newly ingested prey. In comparison to *M. rubrum*, in which full chloroplast turnover took 2–5 wk depending on the concentration of prey offered (Hansen et al. 2012; Peltomaa and Johnson 2017), we observed that in most *M. coatsi* old chloroplasts were replaced by new ones within 4 d when available prey was present in sufficient quantities. Furthermore, no differences in the transition time of chloroplasts were observed when the ciliate switched from *Chroomonas* sp. 09 to *Rhodomonas* sp. 03, or vice versa, both of which have chloroplasts containing different types of pigment (i.e. phycocyanin and phycoerythrin, respectively). This result may imply that *M. coatsi* does not preferentially retain certain chloroplasts for supporting phototrophic growth when both prey types are available. *M. chamaeleon* was also shown to retain different types of chloroplasts by cross‐feeding on different types of cryptophyte prey (Moeller and Johnson 2017); *M. chamaeleon* replaced ~50% of its chloroplasts from *C. mesostigmatica* with those of *Storeatula major* within 2 d, and replaced nearly 100% of these within 8 d.

### Function of retained chloroplasts

When provided cryptophyte prey, *Mesodinium coatsi* was able to undergo heterotrophic growth in darkness, but its growth was significantly enhanced in the presence of light (using a 14:10 h light:dark cycle), i.e. its mixotrophic growth could be supported by either only feeding on prey or by feeding coupled with light (photosynthesis). In particular, light had a significant effect on the positive biomass increase of *M.** **coatsi* (i.e. this was 2.3 times higher in light/dark conditions than in darkness), suggesting that the chloroplasts of its ingested prey are photosynthetically active until being digested. The observed highest growth rate of well‐fed *M.** **coatsi* was 0.55 d^−1^ in light, which was similar to the phototrophic growth of *M.** **rubrum*, which is able to maintain stable photosynthesis for over a month (Hansen and Fenchel 2006; Johnson and Stoecker 2005; Kim et al. 2017; Peltomaa and Johnson 2017). Nonetheless, we also cannot rule out alternate explanations for the effects of light on growth in *M.** **coatsi*. For example, increasing light irradiance often results in the either stimulation of feeding or acceleration of digestion, both of which produce a positive increase in the biomass of protists (Li et al. 1999; Strom 2001; Tarangkoon and Hansen 2011).

In *M. coatsi*, the photosynthetic capacity and stability of the retained prey chloroplasts seems to be somewhat lower than that in *M. rubrum* because we observed that the photosynthetic growth of *M. coatsi* decreased rapidly as soon as prey numbers were depleted. When prey were absent, starved *M. rubrum* was observed to be capable of dividing three to four times and surviving for up to about 4 mo (Johnson and Stoecker 2005; Johnson et al. 2007; Kim et al. 2016, 2017; Nam et al. 2015), whereas in the present study starved *M. coatsi* gradually lost prey chloroplasts more and more with increasing starvation time and survived for only about 2 wks. The difference in photosynthetic ability and survival time between the two species may be associated with differences in which prey organelles are sequestered and how they are packaged within the ciliates. *M. rubrum* has been reported to sequester prey chloroplasts and prey nucleus, which remain transcriptionally active in photosynthetic functions (Johnson et al. 2007; Kim et al. 2016, 2017; Lasek‐Nesselquist et al. 2015), whereas *M. chamaeleon* and *M. coatsi* have been observed to harbor whole cryptophyte cells rather than to sequester certain organelles, and then digest these in food vacuoles within a short time period (Moestrup et al. 2012; Nam et al. 2015). Such a difference in the degree of retention and acquisition of prey organelles may lead the lack of ability of *M. coatsi* to control the prey chloroplasts, unlike *M. rubrum*. Therefore, it appears that *M. coatsi* have to uptake new prey chloroplasts constantly for their continued survival, like *M. chamaeleon* (Moeller and Johnson 2017; Moestrup et al. 2012).

## Conclusions

Our study demonstrated that *Mesodinium coatsi* can use both phototrophic and heterotrophic nutritional strategies. Such a combination of two nutritional modes may allow this ciliate to survive more efficiently in a changing environment. In terms of the categories of mixotrophy recently redefined by Mitra et al. (2016) based on the physiological function of protists, *M.** **coatsi* appears to fall within the “Generalist Non‐Constitutive Mixotroph (GNCM)” functional group. *M.** **coatsi* was able to feed on various types of cryptophytes prey and exploit retained prey chloroplasts, but not for all prey organisms. However, the ability of *M.** **coatsi* to use and control stolen plastids seemed to be poor. Therefore, *M.** **coatsi* appears to continuously seek new chloroplast donors to support its sustained growth. In addition, its photosynthetic ability was similar to that of *M.** **chamaeleon*, but lower than that of *M.** **rubrum*, which implies that *M.** **coatsi* is evolutionarily intermediate between the heterotrophic and phototrophic *Mesodinium* species in terms of its acquired phototrophy, similar to *M.** **chamaeleon* (Moeller and Johnson 2017). This study provides new information to support a better understanding of chloroplast function and status in mixotrophic benthic *Mesodinium* species.

## Supporting information


**Movie S1.** Movie showing that *M. coatsi* captures and feeds on cryptophyte prey *Chroomonas* sp. 07.

**Movie S2.** Movie showing that *M. coatsi* captures and feeds on cryptophyte prey *Chroomonas* sp. 09.

**Movie S3.** Movie showing that *M. coatsi* captures and feeds on cryptophyte prey *Chroomonas* sp. 12.

**Movie S4.** Movie showing that *M. coatsi* captures and feeds on cryptophyte prey *Rhodomonas* sp. 01.

**Movie S5.** Movie showing that *M. coatsi* captures and feeds on cryptophyte prey *Rhodomonas* sp. 02.

**Movie S6.** Movie showing that *M. coatsi* captures and feeds on cryptophyte prey *Rhodomonas* sp. 03.

**Movie S7.** Movie showing that *M. coatsi* captures and feeds on cryptophyte prey *Rhodomonas* sp. 04.

**Movie S8.** Movie showing that *M. coatsi* captures and feeds on cryptophyte prey *Storeatula* sp.

**Movie S9.** Movie showing that *M. coatsi* captures and feeds on cryptophyte prey *Teleaulax amphioxeia*.
