# Supplementary material for: Growth and Chloroplast Replacement of the Benthic Mixotrophic Ciliate Mesodinium coatsi
Source: J Eukaryot Microbiol. 2019 Jan 11;66(4):625–36. doi: 10.1111/jeu.12709 (PMC6766864; doi:10.1111/jeu.12709)
Supplement: Supplementary file 10 [file JEU-66-625-s010.pdf]

SUPPORTING INFORMATION

Growth and Chloroplast Replacement of the Benthic Mixotrophic Ciliate *Mesodinium coatsi* by Miran Kim, Misun Kang, Myung Gil Park

Movie S1. Movie showing that *M. coatsi* captures and feeds on cryptophyte prey *Chroomonas* sp. 07.
Movie S2. Movie showing that *M. coatsi* captures and feeds on cryptophyte prey *Chroomonas* sp. 09.
Movie S3. Movie showing that *M. coatsi* captures and feeds on cryptophyte prey *Chroomonas* sp. 12.
Movie S4. Movie showing that *M. coatsi* captures and feeds on cryptophyte prey *Rhodomonas* sp. 01.
Movie S5. Movie showing that *M. coatsi* captures and feeds on cryptophyte prey *Rhodomonas* sp. 02.
Movie S6. Movie showing that *M. coatsi* captures and feeds on cryptophyte prey *Rhodomonas* sp. 03.
Movie S7. Movie showing that *M. coatsi* captures and feeds on cryptophyte prey *Rhodomonas* sp. 04.
Movie S8. Movie showing that *M. coatsi* captures and feeds on cryptophyte prey *Storeatula* sp..
Movie S8. Movie showing that *M. coatsi* captures and feeds on cryptophyte prey *Teleaulax amphioxeia*.
